# Sacubitril/valsartan reduces cardiac decompensation in heart failure with preserved ejection fraction: a meta-analysis

**DOI:** 10.2459/JCM.0000000000001411

**Published:** 2022-11-15

**Authors:** Christian Basile, Stefania Paolillo, Paola Gargiulo, Federica Marzano, Gaetano Asile, Antonio Luca Maria Parlati, Alfonsina Chirico, Ermanno Nardi, Davide Buonocore, Angela Colella, Pasquale Perrone-Filardi

**Affiliations:** a Department of Advanced Biomedical Sciences, Federico II University of Naples; b IRCCS Synlab SDN S.p.a.; c Mediterranea Cardiocentro, Naples, Italy

**Keywords:** heart failure patients with preserved ejection fractions, meta-analysis, sacubitril/valsartan

## Abstract

**Background:**

The impact of sacubitril–valsartan on heart failure (HF) patients with preserved ejection fractions (HFpEF) is uncertain. The purpose of this meta-analysis was to explore the clinical advantages and safety of sacubitril–valsartan in patients with HFpEF.

**Methods:**

PubMed and Web of Science were searched without any restrictions from inception to 8 May 2022 to identify valuable articles. The studies that met the inclusion criteria were analyzed.

**Results:**

Four trials, with a total of 7008 patients were included. Compared with valsartan, sacubitril–valsartan significantly reduced the rate of HF decompensation and of the combined end point of HF decompensation and all-cause mortality. All-cause mortality, New York Heart Association class improvement and rate of hyperkalemia were not significantly different between the two groups. Regarding safety, sacubitril–valsartan was more likely to increase the risk of hypotension.

**Conclusion:**

This meta-analysis suggests that sacubitril–valsartan may be an effective strategy to reduce HF decompensation events in patients with HFpEF.

**Systematic Review registration:** CRD42022336077.

## Introduction

Heart failure (HF) with preserved ejection fraction (HFpEF) accounts for roughly more than half of symptomatic HF patients and still carries substantial rates of hospitalization and death.^1^ Pathophysiological cornerstones of HFpEF include aberrant diastolic function,^2^ cardiac structural abnormalities, subclinical systolic dysfunction,^3^ and defective natriuretic and renal endocrine responses to acute volume expansion;^4^ affected patients might exhibit several different phenotypes, a condition that complicates the pharmacological approach to this population. While substantial data demonstrated that β-blockers, renin–angiotensin–aldosterone system (RAAS) inhibitors, and angiotensin receptor–neprilysin inhibitors (ARNI) significantly improve the prognosis of HF patients with reduced EF (HFrEF), no consistent data reported a prognostic beneficial role of these classes of drugs in HFpEF. Recent data showed a prognostic impact of Sodium-glucose Cotransporter-2 (SGLT2) inhibitors in HFpEF patients, thus starting to modify the pharmacological approach to HFpEF.^5,6^ As regards ARNI, the PARAGON trial failed for just a few events to demonstrate a prognostic impact of sacubitril/valsartan in HFpEF patients compared with valsartan; moreover, from a combined analysis of PARADIGM-HF and PARAGON-HF trials,^7^ the efficacy of sacubitril/valsartan was evident up to an EF value of 55%, thus including a subgroup of HFpEF patients, with a beneficial effect in women also seen at higher EFs. Apart from the PARAGON trial, some other studies focused on the efficacy of sacubitril/valsartan in HFpEF, even if not specifically designed to analyze its role on major cardiac outcomes. Therefore, the impact of sacubitril–valsartan on patients with HFpEF is still an unsolved issue. This meta-analysis was conducted to explore the possible therapeutic advantages and safety of sacubitril–valsartan in patients with HFpEF (Graphical abstract).

## Methods

This meta-analysis was performed based on the Preferred Reporting Items for Systematic Reviews and Meta Analyses guidelines^8^ (online Table I, Supplemental Digital Content) and registered in PROSPERO (CRD42022331565).

### Patient and public involvement

A patient and public involvement team was not involved in the design, conduct, reporting or dissemination plans of our research. No patients or the public were therefore involved in the present study.

### Search strategy and study selection

PubMed and Web of Science were searched without any restrictions from inception to 8 May 2022. The search strategy is included in the Supplementary Materials, Supplemental Digital Content. Two authors separately examined titles and abstracts of all obtained publications to exclude clearly unrelated research. According to the inclusion criteria, the remaining articles were chosen for full-text examination. The final list of included studies was then reviewed by the authors, and any differences were addressed via discussion. Abstracts presented at international meetings and not followed by indexed publications were not considered. The references list of meta-analyses included in the literature search were reviewed to search additional papers. Studies were included if they satisfied the following criteria: randomized controlled trials (RCTs); individuals treated with sacubitril–valsartan versus valsartan; and studies reporting primary or secondary outcomes.

### Data extraction and quality assessment

The primary efficacy outcome was a composite of all-cause mortality and HF decompensation; secondary efficacy outcomes were all-cause mortality, HF decompensation, and New York Heart Association (NYHA) class improvement. We used the end point of ‘HF decompensation’, instead of the classical end point of HF hospitalization, since the PARAMOUNT and PARALLAX trials did not report data for HF hospitalization, although in the section of adverse events reported the event ‘cardiac failure’, defined in MedDRA^9^ as an HF condition with vary clinical findings, as dependent edema, raised jugular venous pressure, hepatomegaly, pulmonary congestion, tachycardia, cardiomegaly, and dyspnea, all signs, and symptoms of worsening HF. Differently, all studies provided all-cause mortality events. NYHA class improvement was defined as a positive change in NYHA functional class during the study follow-up. As safety outcomes, we analyzed hyperkalemia, defined as a serum potassium level ≥5.5 mmol/l, and hypotension, defined as SBP < 100 mmHg.

Two authors (C.B. and S.P.) independently extracted and compared data, with conflicts pertaining to the source publications addressed by conversation. The following information was gathered from each included study: basic characteristics of studies (authors, publication year, journal, country), patient characteristics (sample size, gender, age, medical history), intervention and control treatments (dose, frequency, duration, mean follow-up time), primary outcomes (risk of HF hospitalization, HF decompensation, cardiovascular mortality), and secondary outcomes (all-cause mortality, improvement of NYHA class, incidence of side effects including hypotension and hyperkalemia). To analyze the risk of bias, the Cochrane Collaboration's tool for assessing risk of bias was used^10^ (online Table II, Supplemental Digital Content).

### Statistical analysis

STATA 17.0 (Stata Corp., College Station, TX, USA) was used to analyze data. The Chi-square test and *I*^2^ test were used to investigate heterogeneity, with *P* ≤ 0.10 or *I*^2^ > 50% indicating considerable heterogeneity. If there was no substantial heterogeneity, risk ratios (RRs) and 95% confidence intervals (CI) were estimated for binary variables using a fixed effect model, otherwise, a random effect model was used. In absence of significant heterogeneity, the weighted mean difference (WMD) and 95% CI were determined for continuous variables, otherwise a random effect model was used. In addition, sensitivity analysis, funnel plots, and Egger's test were performed to assess the stability of estimates and publication bias of included papers. A two-tailed *P*-value of 0.05 was deemed significant.

## Results

### Study characteristics

**Fig. 1:**
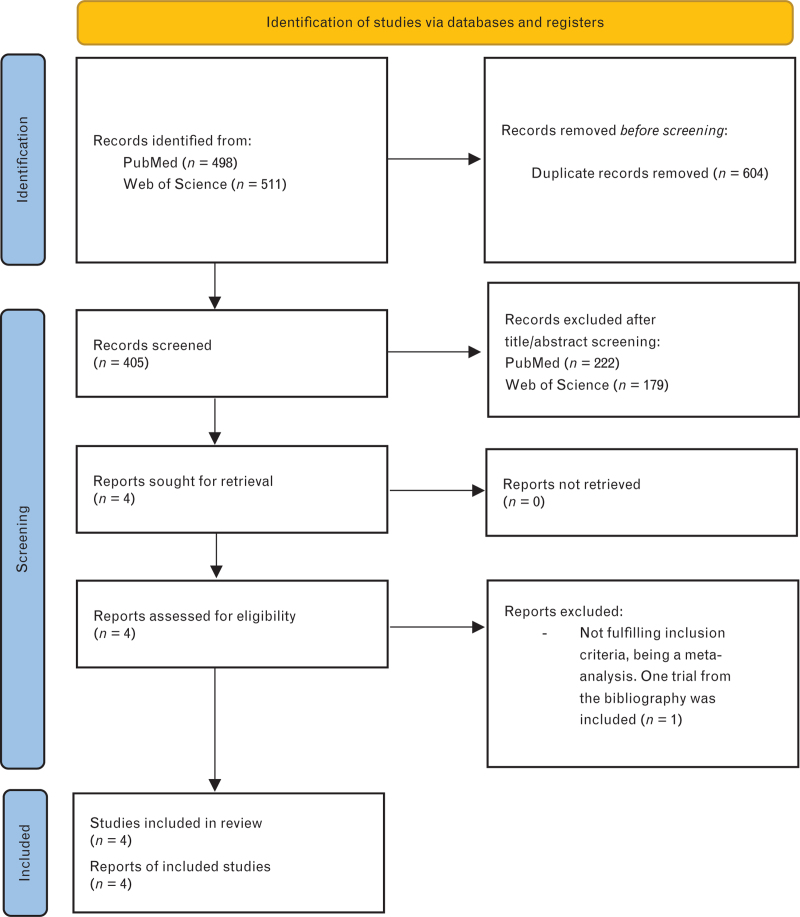
Bibliographic research. PRISMA 2020 flow diagram for new systematic reviews.

Of 405 papers identified in the initial research, four were retrieved for a more detailed evaluation (Fig. 1). According to the inclusion criteria, one study was rejected being a meta-analysis,^11^ but its references list was analyzed finding another trial,^12^ so in total four studies were included,^12–15^ comprising 7008 patients and published between 2012 and 2020. Table 1 provides a summary of the baseline characteristics of the included studies.

**Table 1:** Characteristics of the included studies

Study	Year	Study design	Primary outcome	Inclusion criteria	Follow-up	Total sample size	Sample size included in the final analysis of the trials		Primary outcome (measure of effect, 95% CI)
							ARNI	Valsartan	
PARAMOUNT (NCT00887588)	2012	RCT	Change in plasma NT- proBNP level	-Age ≥40 years-Symptomatic HF- EF ≥45%-Elevated plasma NT-proBNP levels-Diuretic therapy-SBP <140 mmHg or 160 mmHg if on ≥3 antihypertensive drugs-eGFR ≥30 ml/min/1.73 m^2^-Potassium concentration ≤5.2 mmol/l	9 months	301	149	152	Ratio of change: 0.77 (0.64–0.92)
PARAGON (NCT01920711)	2019	RCT	Composite of total HF hospitalizations and death from CV causes	-Age ≥50 years-Signs and symptoms of HF-NYHA class II to IV-EF ≥45% within the previous 6 months-Elevated levels of natriuretic peptides-Evidence of structural heart disease-Diuretic therapy	35 months	4822	2407	2389	RR: 0.87 (0.75–1.01)
Shi *et al.*	2020	RCT	Composite of total HF hospitalizations and death from CV causes	-Age >50 years-Symptomatic HF-NYHA class II-IV-EF ≥50%-NT-proBNP >300 pg/ml, (>900 pg/ml if on atrial fibrillation)	3 months	42	20	22	RR: 0.37 (0.12–1.17)
PARALLAX (NCT03066804)	2020	RCT	Change in plasma NT-proBNP level and in the 6-min walk distance	-Age ≥45 years-Symptomatic HF requiring diuretics-NYHA class II to IV-EF ≥40%-Elevated plasma NT-proBNP levels-Evidence of structural heart disease-KCCQ <75	6 months	1869	1281	588	Adjusted geometric mean ratio estimate: 0.84 (0.80–0.88)

CV, cardiovascular; EF, ejection fraction; eGFR, estimated glomerular filtration rate; HF, heart failure; hs-CRP, high sensitivity C-reactive protein; NYHA, New York Heart Association; KCCQ, Kansas City Cardiomyopathy Questionnaire; RCT, randomized control trial; RR, risk ratio; SBP, systolic blood pressure.

The baseline characteristics – including mean age, sex, basic medical history, NYHA class, left ventricular ejection fraction (LVEF), and baseline treatments for HF – were comparable between sacubitril–valsartan and valsartan groups (Table 2), except for the PARALLAX trial^15^ that had multiple comparators and these characteristics were not available for the valsartan single group. The follow-up varied from 6 to 35 months.

**Table 2:** Characteristics of patients enrolled in the included trials

	Age (years, mean ± SD)		Female (%)		NYHA III class		EF (±SD)		DM		CAD		ACEi or ARBs		Diuretics		Beta-blockers		MRA		SGLT2i	
Study	ARNI	Valsartan	ARNI	Valsartan	ARNI	Valsartan	ARNI	Valsartan	ARNI	Valsartan	ARNI	Valsartan	ARNI	Valsartan	ARNI	Valsartan	ARNI	Valsartan	ARNI	Valsartan	ARNI	Valsartan
PARAMOUNT (NCT00887588)	70.9 (9.4)	71.2 (8.9)	57	56	28	32	58 (7.3)	58 (8.1)	61 (41%)	53 (35%)	32 (21%)	30 (20%)	140 (93.9%)	142 (93.4%)	149 (100%)	152 (100%)	117 (79%)	121 (80%)	28 (19%)	35 (23%)		
PARAGON (NCT01920711)	72.7 (8.3)	72.8 (8.5)	51.6	51.8	458	474	57.6 (7.8)	57.5 (8.0)	1046 (43.5%)	1016 (42.5%)	561 (23.3%)	522 (21.9%)	2074 (86.2%)	2065 (86.4%)	2294 (95.3%)	2291 (95.9%)	1922 (79.9%)	1899 (79.5%)	592 (24.6%)	647 (27.1%)		
Shi *et al.*	68.5 (11.2)	66.7 (13.5)	35	27	11	13	55.3(4.9)	52.7(5.1)	11 (55%)	9 (40.9%)	15 (75%)	14 (63%)										
PARALLAX (NCT03066804)	72.9 (8.4)		50.2		416		56.7 (8.3)	56.2 (8.0)	566 (44.2%)		686 (53.6%)		1115 (87.1%)		1277 (99.8%)		1071 (83.7)		419 (32.7%)		34 (2.7%)	

ACEi, ACE-inhibitors; ARBs, angiotensin receptor blockers; CAD, coronary artery disease; DM, diabetes mellitus; EF, ejection fraction; MRA, mineral-corticoid receptor antagonist; NYHA, New York Heart Association; SGLT2i, sodium-glucose cotransporter 2 inhibitors.

### Efficacy of angiotensin receptor–neprilysin inhibitors in patients with heart failure with preserved ejection fractions

**Fig. 2:**
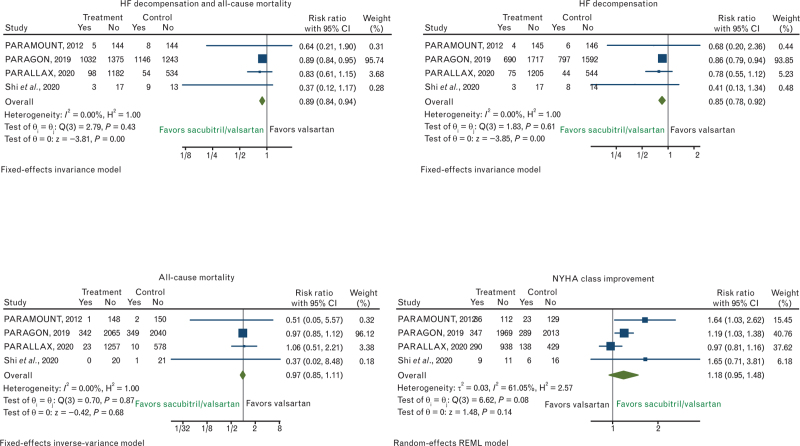
Risk ratios (RRs) for the efficacy outcomes of HF decompensation and all-cause mortality, HF decompensation, all-cause mortality, and NYHA class improvement. Solid squares represent RRs in trials and have a size proportional to the number of events. The 95% confidence intervals (CI) for individual trials are denoted by lines and those for the pooled RRs by empty diamonds. HF, heart failure; NYHA, New York Heart Association.

As regards the primary efficacy outcome of HF decompensation and all-cause mortality, no significant heterogeneity was found (*I*^2^ = 0.00%), hence a fixed effect model was used. Sacubitril–valsartan significantly improved the combined outcome of all-cause mortality and HF decompensation in patients with HFpEF compared with valsartan (RR, 0.89; 95% CI, 0.84–0.94) (Fig. 2). Similarly, as regards the secondary outcomes, no significant heterogeneity was found (*I*^2^ = 0%) for HF decompensation, hence a fixed effect model was used. In the sacubitril–valsartan group, the risk of HF decompensation was considerably lower than in the valsartan group (RR, 0.85; 95% CI, 0.78–0.92) (Fig. 2). Investigating all-cause mortality, no significant heterogeneity was found (*I*^2^ = 0%), hence a fixed effect model was used, although sacubitril–valsartan did not significantly improve all-cause mortality of HFpEF patients compared to valsartan (RR, 0.97; 95% CI, 0.85–1.11) (Fig. 2). The improvement of NYHA class showed substantial heterogeneity (*I*^2^ = 61.05%), hence a random-effect model was used and no significant difference between sacubitril–valsartan and valsartan was observed for this reported secondary outcome (RR, 1.18; 95% CI, 0.95–1.48) (Fig. 2).

### Safety of angiotensin receptor–neprilysin inhibitors in patients with heart failure with preserved ejection fractions

**Fig. 3:**
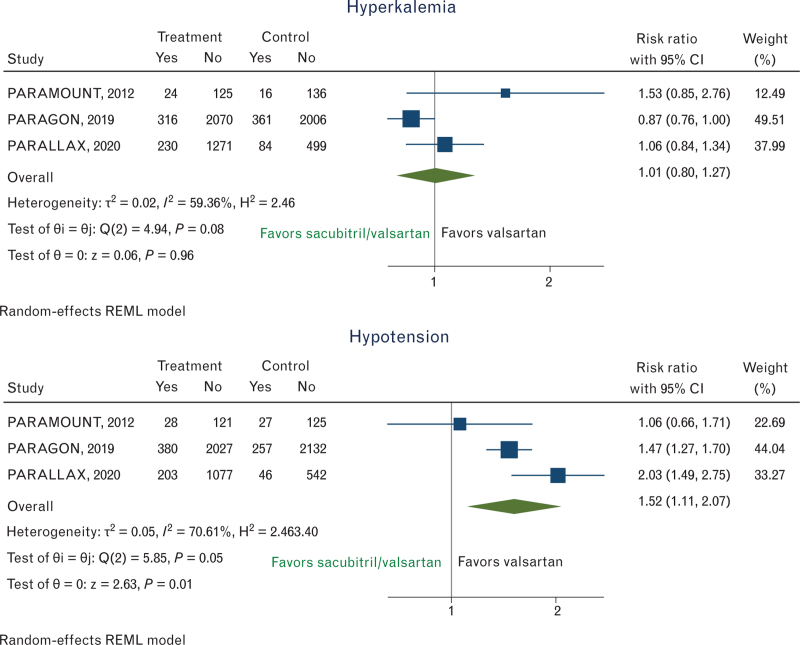
Risk ratios (RRs) for the safety outcomes of hypotension and hyperkalemia. Solid squares represent RRs in trials and have a size proportional to the number of events. The 95% confidence intervals (CI) for individual trials are denoted by lines and those for the pooled RRs by empty diamonds.

The risk of hyperkalemia showed substantial heterogeneity (*I*^2^ = 59.36%) and no significant differences between sacubitril–valsartan and valsartan groups (RR, 1.01; 95% CI, 0.80–1.27) (Fig. 3), while there was a significantly increased risk of hypotension in the sacubitril–valsartan group vs. valsartan (RR 1.52; 95% CI, 1.11–2.07) (Fig. 3), although in the presence of significant heterogeneity (*I*^2^ = 70.61%) among studies.

### Publication bias and sensitivity analysis

Due to the low number of studies, publication bias for all outcomes was assessed. Funnel plots (online Figure I, Supplemental Digital Content) and Egger's tests revealed no publication bias (online Table III, Supplemental Digital Content), with the exception of the incidence of hyperkalemia. To evaluate the consistency of the findings, sensitivity analyses were conducted on all outcomes (online Figure II, Supplemental Digital Content).

## Discussion

The present meta-analysis suggests that sacubitril–valsartan, compared with valsartan, lowers the risk of the composite outcome of HF decompensation and all-cause mortality in patients with HFpEF, followed by a significantly reduced risk of HF decompensation. Neither all-cause mortality nor NYHA class improves considerably, nor does the occurrence of hyperkalemia, while the likelihood of hypotension significantly increases with sacubitril–valsartan.

In contrast to HFrEF patients, the pharmacological treatment for HFpEF is still challenging. As regards RAAS inhibitors, none of the large RCTs conducted in HFpEF has achieved their primary end points, such as PEP-CHF (perindopril),^16^ CHARM-Preserved (candesartan),^17^ although they showed a reduction in HF hospitalizations, I-PRESERVE (irbesartan),^18^ and TOPCAT (spironolactone)^19^ trials. The subgroup of patients in the TOPCAT study enrolled in the US had a significant reduction in the primary end point of CV death and HF hospitalization, and a subsequent posthoc analysis by EF showed a significant reduction in HF hospitalizations for those with an LVEF <55%, with a similar trend for cardiovascular (CV) but not all-cause mortality. Recent trials with SGLT2 inhibitors showed a beneficial effect of these drugs in HFpEF,^5,20^ thus providing relevant changes in the therapeutic approach to this condition to be applied in the near future.^1,21^ As regards the combined RAAS and neprilysin inhibition, sacubitril–valsartan failed to demonstrate a beneficial effect in HFpEF on the combined end point of CV mortality and total HF hospitalizations in the PARAGON-HF trial, although a trend towards a reduction in HF hospitalizations was observed. However, subgroup analysis from the PARAGON-HF trial reported a reduction in HF hospitalizations in patients with an EF <57% and a pooled analysis of the PARADIGM-HF and PARAGON-HF studies showed a reduction in CV death and HF hospitalization in those with an EF below the normal range.^7^ Thus, the Food and Drug Administration (FDA) has endorsed the use of sacubitril/valsartan and spironolactone in patients with an EF ‘less than normal’, according to clinical judgment. In this complex therapeutic panorama, the practical use of sacubitril–valsartan in HFpEF is still not defined, still not uniform among countries, and related to local authorizations, since its beneficial effects in this context have not been clearly elucidated by available trials.

In the present meta-analysis, we tried to analyze data from the three major studies assessing the effects of ARNI in HFpEF, namely the PARAMOUNT, PARALLAX and PARAGON-HF trials, even if only the latter was specifically designed to evaluate the role of sacubitril–valsartan on major CV outcome, whereas the others focused on soft end points, although reporting safety data on clinical cardiac events. Moreover, differently from a previous similar meta-analysis on the topic published as a research letter,^22^ we added a trial published in 2020 and analyzing similar end points. Moreover, in this mentioned previous meta-analysis^22^ all patients enrolled in the PARALLAX trial were included, whereas we specifically focused on valsartan as a comparator, thus excluding from the analysis PARALLAX patients not treated with valsartan but with other comparators (enalapril, placebo).

Starting from these data, we observed a significant reduction in the composite outcome of all-cause mortality and HF decompensation compared with valsartan (RR, 0.89; 95% CI, 0.84–0.94) (Fig. 2), with considerably lower risk of HF decompensation (RR, 0.85; 95% CI, 0.78–0.92) (Fig. 2) and no effects on all-cause mortality (RR, 0.97; 95% CI, 0.85–1.11) (Fig. 2). Differently from the commonly used end point of HF hospitalization, in this analysis we used the component ‘HF decompensation’, due to the absence of data for HF hospitalization in the PARAMOUNT and PARALLAX trials that however included ‘cardiac failure’ events in the section of adverse events. Even if this approach might be considered as a limitation of the present analysis introducing a bias that must be considered, on the other hand expands the study of the effects of sacubitril–valsartan on a wider HF end point, including not only hospitalizations but also other types of HF decompensation, not necessarily accompanied by hospitalization. HF decompensation can occur with several different clinical presentations and levels of gravity, and not all worsening HF events require hospitalization; nevertheless, in reality, HF hospitalization is not always quickly accessible, thus many of these patients after an urgent admission in the HF outpatient service are then home managed. Thus, an HF disease-modifier drug should desirably act on all manifestations of decompensation since all forms of decompensation are adversely related to a decline in cardiac function and to disease progression. The results of this meta-analysis might be considered hypothesis generating findings; the PARAGON-HF trial failed for a few events to meet the primary end point, although a trend towards reduction of HF hospitalization was observed, confirmed in patients with EF <57% in subgroup analysis. Thus, the inclusion of a wider end point of HF events might emphasize the potential beneficial effect of sacubitril–valsartan in HFpEF, as was done for other disease-modifiers, such as dapagliflozin in HFpEF in the DELIVER trial (NCT03619213) that considered as the primary end point a composite of CV mortality or worsening HF events.^20^

As regards safety analysis, in patients with HFpEF, hypotension occurred more often in patients taking sacubitril–valsartan, an observation consistent with prior studies, although, sacubitril–valsartan was not more likely to cause significant hyperkalemia.

There were several limitations in this meta-analysis. First, the low number of clinical trials and small sample sizes of single trials may have introduced bias into the estimations. Second, it was not feasible to assess sacubitril–valsartan effects on NT-proBNP changes or quality of life, assessed by Kansas City Cardiomyopathy Questionnaire, to preserve consistency with the variables studied, since these data were not provided in all publications. Third, the different follow-up between the trials may have reduced the ability to better estimate the effect of sacubitril–valsartan in the long term in these patients.

## Conclusion

In summary, this study suggests that, compared with valsartan, sacubitril–valsartan may lower the risk of a composite outcome of HF decompensation and all-cause mortality in patients affected by HFpEF, preferably acting on a significant reduction in the risk of HF decompensation events. Additional, well designed RCTs are required in the near future to validate these results and to definitively determine if sacubitril–valsartan has unique advantages in patients with HFpEF.

### Conflicts of interest

There are no conflicts of interest.

## Supplementary Material
