# Supplementary material for: Sacubitril/valsartan reduces cardiac decompensation in heart failure with preserved ejection fraction: a meta-analysis
Source: J Cardiovasc Med (Hagerstown). 2022 Nov 15;24(1):44–51. doi: 10.2459/JCM.0000000000001411 (PMC9794138; doi:10.2459/JCM.0000000000001411)

Graphical abstract

Title: Sacubitril/valsartan in heart failure with preserved ejection fraction: Efficacy and safety

Caption: This figure shows the meta-analysis in summary and the main findings on the composite of HF decompensations and all-cause mortality, HF decompensation, hyperkalemia and hypotension. HF, heart failure.


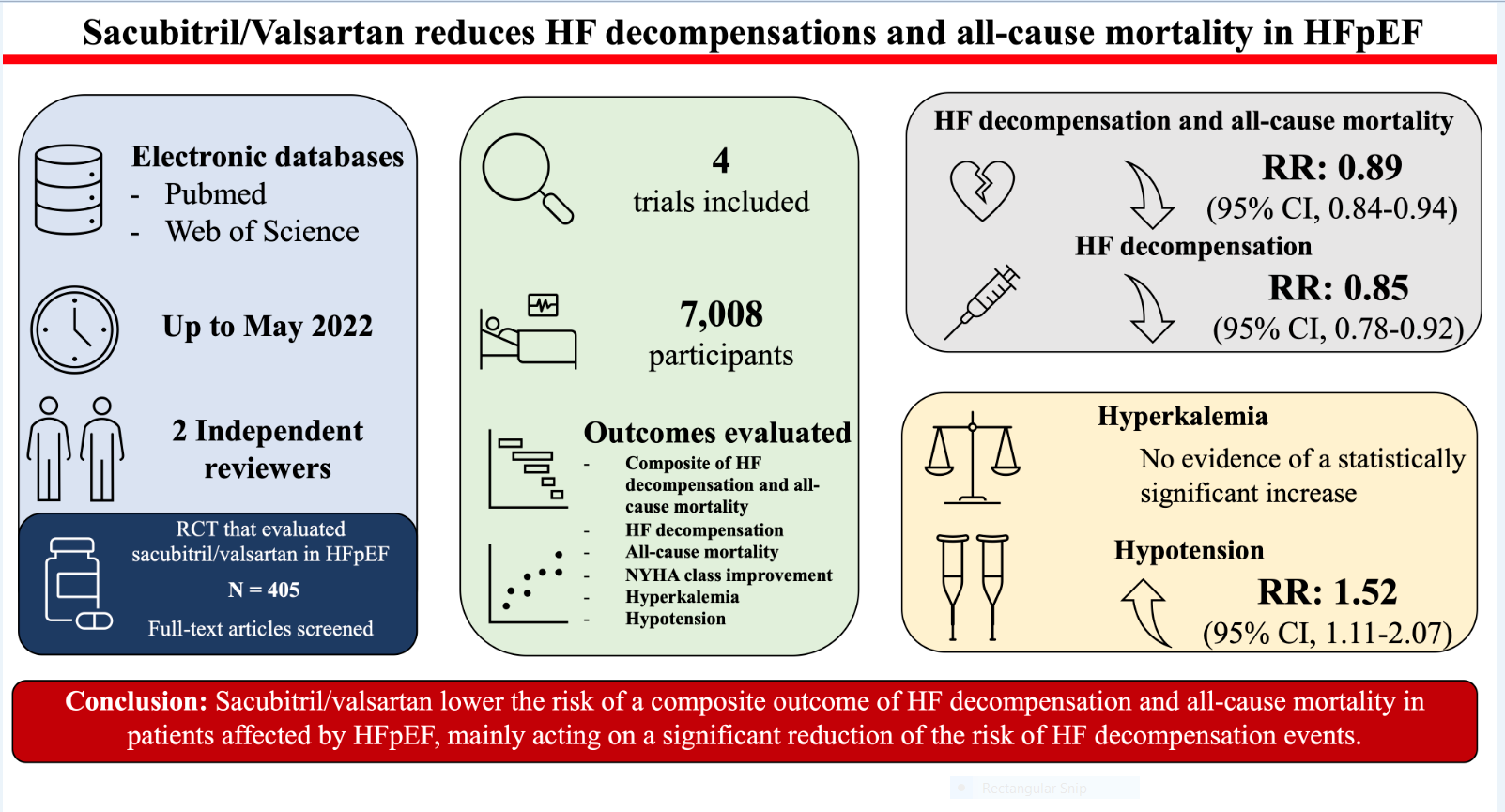

Supplement: Supplementary file 1 [file jcarm-24-44-s001.doc]
